# LINC01770 Is Associated with Stem-like Features and Aggressive Traits in Breast Cancer Cells Through a Putative miR-335-5p/OCT4 Axis

**DOI:** 10.3390/ph19071039

**Published:** 2026-07-03

**Authors:** Javier Gasson, Antonia Böhmwald, Juan P. Muñoz, Mauricio A. Retamal, Pablo Pérez-Moreno

**Affiliations:** 1 Programa de Comunicación Celular en Cáncer, Instituto de Ciencias e Innovación en Medicina (ICIM), Facultad de Medicina, Clínica Alemana Universidad del Desarrollo, Santiago 7780272, Chile; jgassonz@udd.cl (J.G.); abohmwaldv@udd.cl (A.B.); mretamal@udd.cl (M.A.R.); 2 Laboratorio de Bioquímica, Departamento de Química, Facultad de Ciencias, Universidad de Tarapacá, Arica 1000007, Chile; jpmunozb@academicos.uta.cl

**Keywords:** LINC01770, breast cancer, stem-like phenotype

## Abstract

**Background/Objectives:** Long non-coding RNAs (lncRNAs) are regulatory transcripts that contribute to diverse cellular processes and are increasingly recognized for their involvement in human diseases including cancer. In this context, long intergenic non-protein-coding RNA 1770 (LINC01770), also known as RRFERV, has been involved in nasopharyngeal cancer progression. However, its role in breast cancer (BC) remains unexplored. Here, we propose that LINC01770 plays a pivotal role in the development of aggressiveness traits such as invasion, migration, stemness, and tumorigenesis in BC cells. **Methods:** The LINC01770 overexpression was performed in BC cells using lentiviral transduction. Stemness and epithelial–mesenchymal transition markers, CD133^+^/44^+^ populations, cell migration, cell invasion, tumorigenesis in vitro, and chemoresistance were subsequently assessed via quantitative reverse transcription polymerase chain reaction (RT-qPCR), flow cytometry, Boyden chambers, soft agar, and 3-(4,5-dimethylthiazol-2-yl)-5-(3-carboxymethoxyphenyl)-2-(4-sulfophenyl)-2H-tetrazolium (MTS) assays, respectively. LINC01770 expression in BC tissues and mechanistic analyses were performed in silico. **Results:** LINC01770 promotes cell migration and invasion accompanied by increased expression of EMT-associated genes. Moreover, elevated LINC01770 levels lead to an expansion of CD133^+^/CD44^+^ cell populations and upregulation of stemness-related genes as well as increase tumorigenic capacity in vitro. In contrast, no significant effects on drug resistance were observed. Finally, bioinformatic analyses suggest a putative LINC01770/miR-335-5p/OCT4 regulatory axis, consistent with the observed increase in OCT4 expression after LINC01770 overexpression. **Conclusions:** Our findings demonstrate that LINC01770 drives BC progression by promoting migration, invasion, and stemness features via the miR-335-5p/OCT4 axis. To our knowledge, this is the first study identifying LINC01770 as a potential therapeutic target in BC.

## 1. Introduction

Breast cancer (BC) represents the most prevalent malignancy and the primary cause of cancer-associated deaths in the female population globally, accounting for over 2.2 million new cases and approximately 666,000 deaths annually [1]. Its clinical heterogeneity encompasses distinct molecular subtypes with variable prognosis and therapeutic responses [2]. Despite advances in early detection and systemic therapies, a significant proportion of patients still experience disease progression, metastasis, and treatment resistance, underscoring the need to better understand the molecular mechanisms that drive BC aggressiveness and therapeutic outcome [3].

While cancer research has traditionally centered on protein-coding genes, growing evidence has revealed that non-coding RNAs (ncRNAs) play essential regulatory roles in tumor biology. These include microRNAs (miRNAs), small nuclear RNAs (snRNAs), P-element-induced wimpy testis (PIWI)-interacting RNAs (piRNAs), and long non-coding RNAs (lncRNAs) [4]. lncRNAs have been shown to play an active role in the regulation of gene expression, thereby contributing to the acquisition of more aggressive tumor phenotypes [5]. For instance, metastasis-associated lung adenocarcinoma transcript 1 (MALAT1) has been reported to promote proliferation, invasion, and metastasis of BC cells through the MALAT1/miR-106a-5p/REEP5 regulatory axis [6]. Another example is small nucleolar RNA host gene 1 (SNHG1), whose downregulation inhibits BC cell proliferation, migration, invasion, epithelial–mesenchymal transition (EMT), and tumor growth in vivo via the SNHG1/miR-641/RRS1 axis, suggesting that SNHG1 may represent a potential therapeutic target for BC [7].

Despite the growing number of lncRNAs implicated in cancer progression, a large proportion of these transcripts remain functionally uncharacterized. Based on this, many lncRNAs are expressed in BC cells and their contribution to tumor aggressiveness and cellular plasticity has not been systematically explored [8]. LINC01770, also known as RRFERV, has been reported in nasopharyngeal carcinoma (NPC), particularly in radiation-resistant cells, and is associated with poor prognosis [9]. Mechanistically, this lncRNA stabilizes TEA domain transcription factor 1 (TEAD1) by sponging microRNA-615-5p and microRNA-1293, thereby promoting malignant progression and radioresistance in NPC [9]. Moreover, the RRFERV–TEAD1 signaling axis plays a role in modulating lipid peroxidation in radiation-resistant NPC cells through the activation of acyl-CoA synthetase long-chain family member 4 (ACSL4) and transferrin receptor (TFRC) [9]. Given its role in radioresistance in other malignancies, LINC01770 emerges as a prime candidate for driving cellular plasticity and aggressive phenotypes in BC.

The present study provides the first functional characterization supporting a pro-tumorigenic role for LINC01770 in BC. Specifically, we aimed to elucidate the contribution of LINC01770 to the acquisition of aggressive phenotypic traits in BC cells.

## 2. Results

### 2.1. LINC01770 Expression in Breast Cancer Tissues

**Figure 1:**
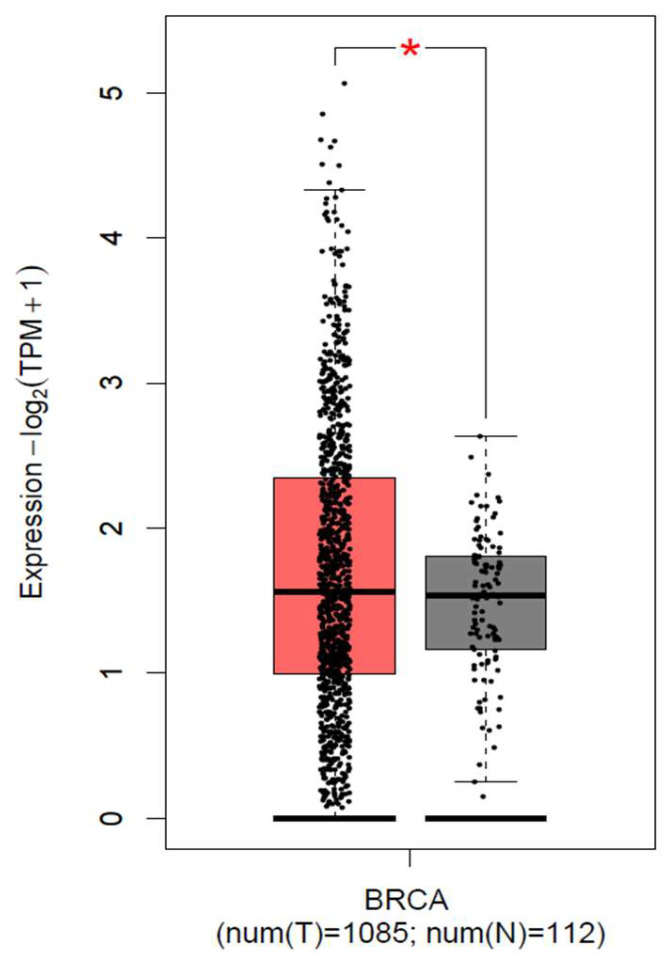
LINC01770 expression profile in human BC cohorts. LINC01770 expression levels in BC tissues (num(T), tumor; red box) compared to normal breast tissues (num(N), normal; gray box) in breast cancer patients (BRCA) derived from the TCGA dataset via the GEPIA2 platform. Boxplots represent expression levels calculated as Log_2_ Transcripts Per Million (TPM + 1). Statistical significance was determined by one-way Analysis of Variance (ANOVA). * *p* ≤ 0.05.

To evaluate the clinical relevance of LINC01770 in human BC, we analyzed its expression profile from the TCGA dataset using the GEPIA2 platform. LINC01770 transcript levels were compared between BC tissues and adjacent normal breast tissues. The bioinformatic analysis revealed a statistically significant upregulation of LINC01770 in tumor samples compared to their respective normal counterparts (Figure 1).

### 2.2. Baseline Expression of LINC01770 and Lentiviral Transduction Efficiency in Breast Cancer Cell Lines

**Figure 2:**
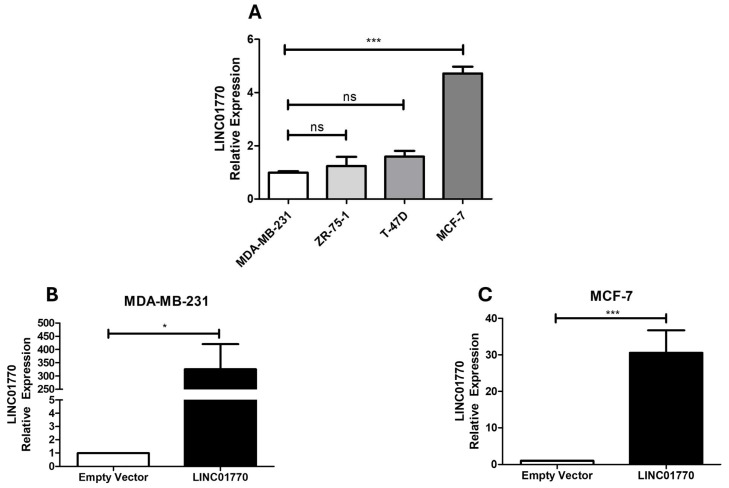
Basal expression of LINC01770 and validation of lentiviral overexpression in breast cancer cell lines. The total RNA obtained was converted into cDNA to perform qPCR for measuring LINC01770 levels. (**A**) Higher LINC01770 expression was observed in the MCF-7 cell line. Conversely, MDA-MB-231 showed lower expression between cell lines. (**B**) MDA-MB-231 and (**C**) MCF-7 cells showed successful overexpression of LINC01770 following lentiviral transduction. Data represents the mean of three independent experiments. ANOVA and Tukey’s post-test were used for Figure 2A. The *t*-test was used for Figure 2B,C. * *p* ≤ 0.05, *** *p* ≤ 0.001; ns: not significant.

The basal expression of LINC01770 was assessed in different BC cell lines by quantitative reverse transcription polymerase chain reaction (RT-qPCR). MDA-MB-231 exhibited the lowest LINC01770 expression, whereas MCF-7 showed the highest levels (Figure 2A). ZR-75-1 and T-47D displayed expression levels similar to MDA-MB-231 but demonstrated slow growth rates and poor transduction efficiencies, making them unsuitable for transduction and phenotypic experiments. Based on these results, MDA-MB-231 and MCF-7 were selected as models for LINC01770 overexpression studies (Figure 2A). LINC01770 was efficiently overexpressed, achieving an approximate 230-fold increase in MDA-MB-231 cells and an approximate 30-fold increase in MCF-7 cells compared to empty vector control cells as confirmed by RT-qPCR (Figure 2B,C). These data validate the suitability of these two cell lines for downstream functional studies of LINC01770.

### 2.3. LINC01770 Promotes Migration, Invasion, and the Expression of Epithelial–Mesenchymal Transition Genes in BC Cell Lines

**Figure 3:**
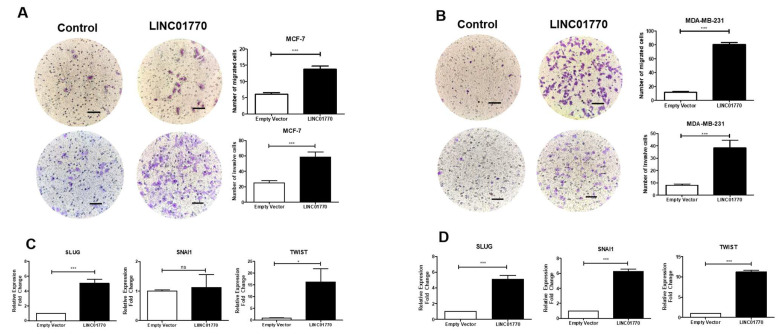
LINC01770 overexpression showed increased migration and invasion capacities in breast cancer cells. LINC01770 overexpression promoted migration and invasion in (**A**) MCF-7 and (**B**) MDA-MB-231 cell lines. Meanwhile, LINC01770 overexpression promoted EMT-associated gene expression in (**C**) MCF-7 and (**D**) MDA-MB-231 cell lines. Magnification of representative images is 40×; scale bar = 100 µm. Data represents the mean of three independent experiments. The *t*-test was used. * *p* ≤ 0.05 and *** *p* ≤ 0.001; ns: not significant.

The migratory and invasive capacities of tumor cells are key features associated with metastatic dissemination [10]. To this end, a migration and invasion assay showed that LINC01770 overexpression promotes greater migration and invasion capacity in MCF-7 and MDA-MB-231 cell lines (Figure 3A,B), upregulating the expression of EMT-related genes such as *SLUG*, *SNA1*, and *TWIST* (Figure 3C,D). To exclude the possibility that the increased number of migrated or invaded cells was due to differences in cell proliferation, we also evaluated proliferation kinetics in both cell lines. No significant differences in proliferation were observed up to 72 h between LINC01770-overexpressing and control cells (Figure S1), indicating that the enhanced migration and invasion are not attributable to increased cell growth.

### 2.4. LINC01770 Promotes Anchorage-Independent Growth in BC Cells

**Figure 4:**
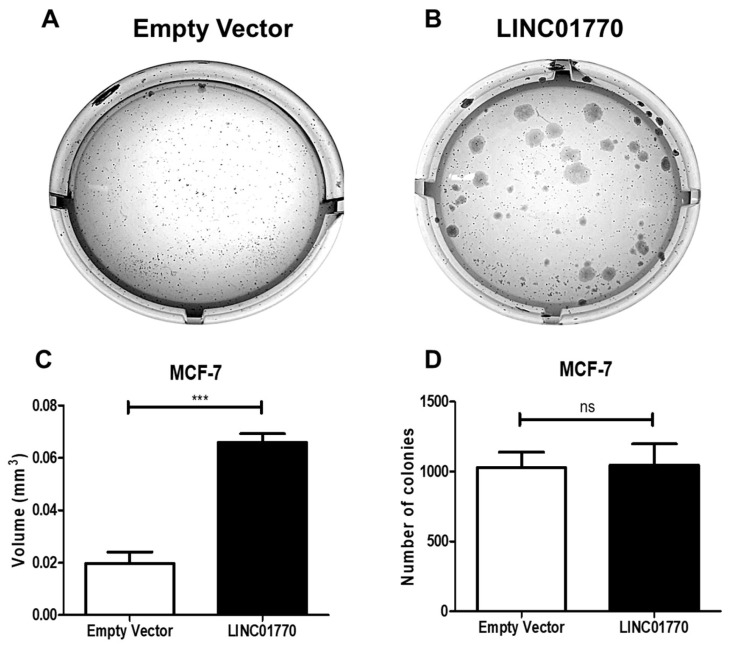
LINC01770 overexpression promotes tumorigenesis in vitro in breast cancer cells. Representative full-field images of wells show anchorage-independent growth in soft agar for (**A**) control and (**B**) LINC01770-overexpressing cells. Quantification of (**C**) colony volume and (**D**) colony number. Data represents the mean of three independent experiments. The *t*-test was used. *** *p* ≤ 0.001; ns, not significant.

To determine whether LINC01770 promotes tumorigenic capacity in vitro, we evaluated anchorage-independent growth using a soft agar assay (Figure 4A,B). MDA-MB-231 cells did not form colonies under these conditions regardless of LINC01770 expression status. In contrast, LINC01770 overexpression in MCF-7 cells markedly increased colony volume (Figure 4C); however, no significant differences were observed in the number of colonies (Figure 4D). This indicates that LINC01770 does not necessarily increase the frequency of colony-initiating cells but significantly enhances the proliferative potential and survival of established clones under anchorage-independent conditions.

### 2.5. LINC01770 Promotes CD133^+^/CD44^+^ Cell Population in BC Cells

**Figure 5:**
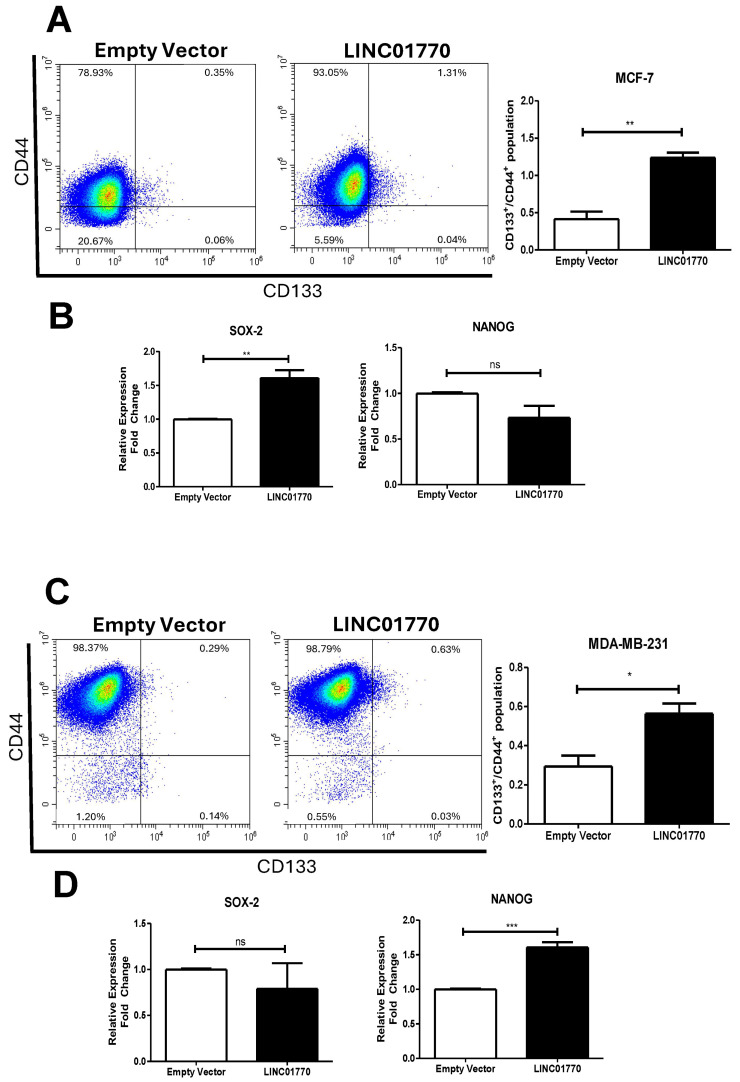
LINC01770 promotes stem-like phenotype in breast cancer cell lines. Overexpression of LINC01770 in (**A**) MCF-7 and (**C**) MDA-MB-231 BC cells increased CD133^+^/CD44^+^ population. (**B**) MCF-7 and (**D**) MDA-MB-231 cells showed higher gene expression of transcription factors associated with stem-like phenotype after LINC01770 overexpression. Data represents the mean of three independent experiments. The *t*-test was used. * *p* ≤ 0.05, ** *p* ≤ 0.01, *** *p* ≤ 0.001; ns: not significant.

The acquisition of a stem-like phenotype has been linked to increased tumor aggressiveness [11]. Cells exhibiting this phenotype are characterized by the upregulation of core stemness-associated transcription factors such as NANOG, SOX2, and OCT4 alongside surface markers, including CD133 (prominin-1) and CD44. [12]. Our results showed that elevated LINC01770 expression increases the proportion of CD133^+^/CD44^+^ cells (Figure 5A,C) and enhances the expression of key transcription factors associated with stem-like properties (Figure 5B,D).

### 2.6. LINC01770 Does Not Promote Resistance to 5-Fluorouracil and Cisplatin in BC Cells

**Figure 6:**
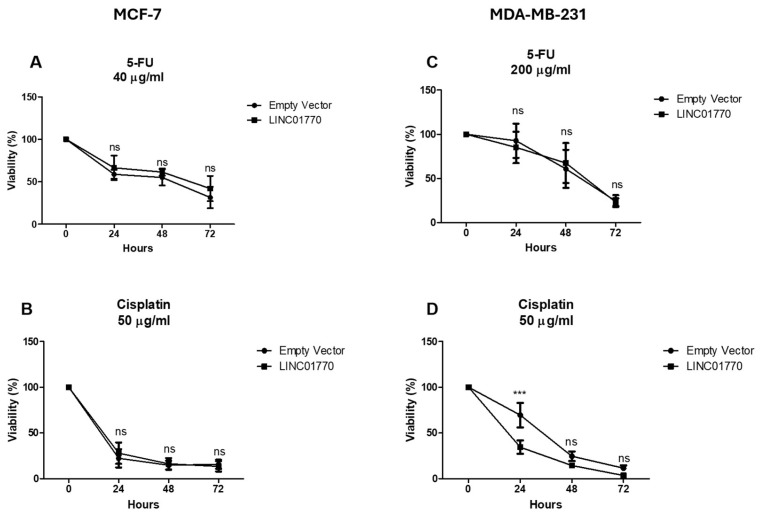
LINC01770 did not show improvement in chemoresistance to 5-fluorouracil and cisplatin in breast cancer cells. (**A**,**B**) MCF-7 and (**C**,**D**) MDA-MB-231 cells did not show an increase in cell viability upon exposure to cisplatin and 5-FU treatments after LINC01770 overexpression compared to the control group. Data represents the mean of three independent experiments. ANOVA and Tukey’s post-test were used. *** *p* ≤ 0.001; ns: not significant.

To evaluate whether LINC01770 modulates chemoresistance, MDA-MB-231 and MCF-7 BC cell lines were exposed to different concentrations of 5-fluorouracil (5-FU) and cisplatin to determine optimal working concentrations (Figure S2). Based on these dose–response profiles, MCF-7 cells were treated using 40 µg/mL 5-FU and 50 µg/mL cisplatin. For MDA-MB-231 cells, the concentrations were 200 µg/mL 5-FU and 50 µg/mL cisplatin. Interestingly, despite the significant increase in cancer stem cell (CSC) markers described in previous sections, LINC01770 overexpression did not significantly alter cell viability in response to 5-FU or cisplatin treatments in either cell line (Figure 6). These results suggest that, under the experimental conditions tested, LINC01770 promotes aggressive phenotypic traits without conferring increased resistance to 5-FU or cisplatin.

### 2.7. LINC01770 Induces Cell Aggressiveness via the LINC01770/miR-335-5p/OCT4 Axis

**Figure 7:**
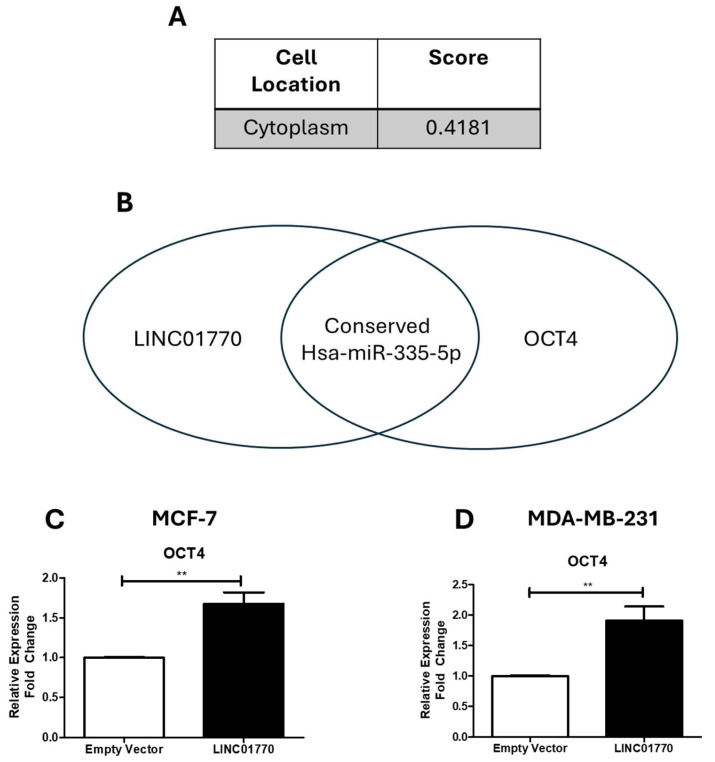
The LINC01770/miR-335-5p axis in breast cancer cells. (**A**) The localization score of LINC01770 indicates that this lncRNA is predominantly compartmentalized within the cytoplasm. (**B**) Venn diagram based on in silico analyses illustrating the predicted interactions among LINC01770, miR-335-5p, and *OCT4*. (**C**,**D**) The upregulation of LINC01770 enhanced the expression of the stemness marker OCT4 in MCF-7 and MDA-MB-231 cells. Data represents the mean of three independent experiments. The *t*-test was used. ** *p* ≤ 0.01.

Our results described above demonstrated that LINC01770 overexpression expands the CD133^+^/CD44^+^ cell populations and increases the expression of stemness-associated transcriptional programs in breast cancer cells. To explore a potential molecular mechanism underlying these effects, we performed in silico analyses focused on the possible interaction among LINC01770, miR-335-5p, and OCT4. First, to determine its subcellular distribution and regulatory relationship with stemness, computational modeling predicted a predominantly cytoplasmic localization for LINC01770 with a high confidence score of 0.4181 (Figure 7A), suggesting its potential role as a competing endogenous RNA. Based on this, subsequent predictive screening using TargetScan to identify miRNAs targeting the core stemness genes revealed that miR-335-5p, a well-characterized tumor suppressor [13,14], harbors conserved binding sites specifically for OCT4 (Figure 7B). Interestingly, in silico predictions identified hsa-miR-335-5p as a downstream target of LINC01770, suggesting that this lncRNA may negatively regulate the miRNA to favor increased OCT4 expression (Figure 7B). Consistent with this predicted axis, RT-qPCR analysis showed that LINC01770 overexpression significantly increases OCT4 mRNA levels in both MCF-7 and MDA-MB-231 cells (Figure 7C,D).

Together, these bioinformatic results support a putative model in which LINC01770 may modulate OCT4 expression through an miR-335-5p-related mechanism.

### 2.8. LINC01770 Induces Cell Aggressiveness via the Putative LINC01770/miR-335-5p/OCT4 Axis

**Figure 8:**
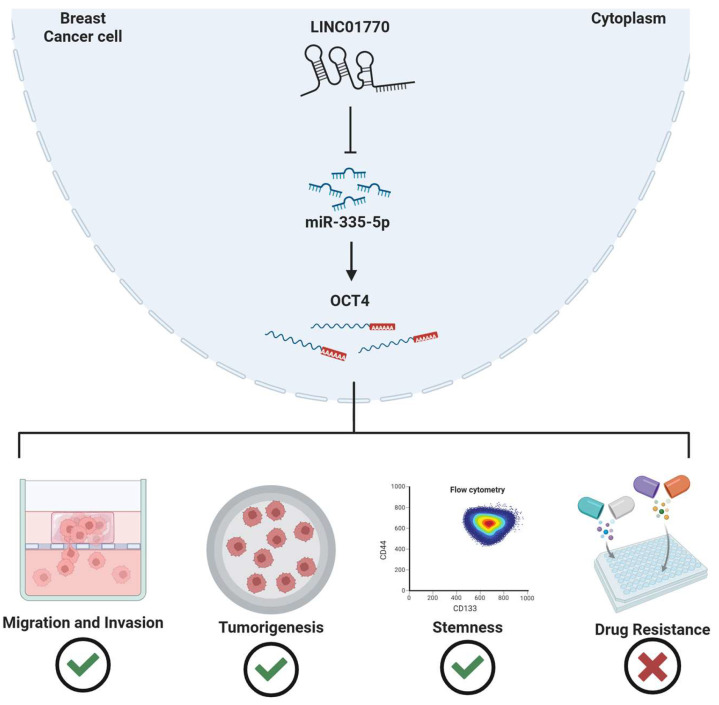
Schematic summary of the putative regulatory mechanism. Schematic summary of the putative LINC01770/miR-335-5p/OCT4 regulatory axis. Graphical representation of the proposed model by which LINC01770 may contribute to stem-like features, migration, and invasion in breast cancer cell lines through a predicted miR-335-5p/OCT4-related mechanism, where LINC01770 acts as a competitive endogenous RNA to sponge miR-335-5p, leading to the upregulation of OCT4 expression.

Cytoplasmic LINC01770 is predicted to act as a competitive endogenous RNA by competitively sponging the tumor suppressor miR-335-5p. This putative interaction prevents the miRNA-mediated repression of OCT4 leading to its subsequent upregulation. Consequently, enhanced OCT4 expression drives the expansion of stem-like cell populations and accelerates cell migration and invasion, ultimately promoting tumor aggressiveness in BC (Figure 8).

## 3. Discussion

In recent years, interest in lncRNAs as potential biomarkers of tumor aggressiveness has increased substantially. This growing attention stems from their active involvement in key cellular processes that drive malignant progression including the modulation of transcriptional programs, signaling pathways, and cellular plasticity [11]. In this study, we provide the first functional characterization of LINC01770 in cancer and demonstrate its contribution to the acquisition of aggressive traits in BC cells.

First, the selection of BC cell lines for LINC01770 overexpression was based on basal expression levels and experimental feasibility aiming to represent the molecular heterogeneity of BC. Based on our results, MDA-MB-231 cells were selected as an optimal model due to their low endogenous LINC01770 expression, which provides a suitable background to evaluate gain-of-function effects. In contrast, MCF-7 cells exhibited higher basal levels of LINC01770. However, they were successfully transduced achieving robust overexpression and consistent modulation of functional phenotypes. Importantly, despite the presence of higher endogenous expression, LINC01770 overexpression in MCF-7 cells was sufficient to recapitulate the functional effects observed in MDA-MB-231 cells, including the induction of stem-like features, migration, invasion, and tumorigenesis features (Figure 3, Figure 4 and Figure 5). Despite the marked differences in basal expression levels between models MCF-7 (luminal A) and MDA-MB-231 (triple-negative), LINC01770 overexpression consistently promoted aggressive functional changes in both cell lines (Figure 2). This suggests that the pro-tumorigenic impact of LINC01770 is not restricted to low-expression contexts and supports the robustness and reproducibility of its functional role across distinct BC cellular backgrounds. However, it is important to acknowledge certain limitations. While our gain-of-function approach successfully demonstrates that LINC01770 overexpression drives aggressive phenotypic changes across these distinct backgrounds, a complementary loss-of-function strategy was not performed. Conducting knockdown experiments, particularly in MCF-7 cells which exhibit higher baseline endogenous levels of this lncRNA, would provide valuable insights into its physiological necessity in non-overexpressed systems. This loss-of-function characterization represents a crucial milestone for our future follow-up investigations into the regulatory networks of LINC01770.

LINC01770 overexpression enhanced migratory and invasive capacities and was accompanied by the upregulation of genes associated with EMT (Figure 3). EMT is a well-established mechanism underlying tumor cell dissemination and is tightly interconnected with stemness acquisition and cellular plasticity [15]. The coordinated increase in EMT-associated gene expression and invasive behavior observed in LINC01770-overexpressing cells supports the notion that this lncRNA contributes to aggressive tumor phenotypes through the modulation of interconnected stemness and EMT programs.

LINC01770 expression significantly increases CD133^+^/CD44^+^ cell populations and upregulates key transcription factors associated with stemness (Figure 5). The enrichment of these cell populations is widely recognized as a hallmark of cancer stem-like cells and has been linked to tumor initiation, metastatic potential, and disease recurrence [16,17]. Consistent with previous reports describing oncogenic lncRNAs such as MALAT1 and hox transcript antisense RNA (HOTAIR) as regulators of stemness-related programs [18,19], our findings suggest that LINC01770 functionally aligns with this group of lncRNAs by promoting a stem-like phenotype in BC cells.

As previously established, LINC01770 promotes both EMT and stemness, reinforcing the well-documented link between these two processes in driving tumor aggressiveness [20,21,22]. Our findings are consistent with previous reports suggesting that the convergence of mesenchymal traits and stem-like properties is a critical driver of malignancy in BC cells [23]. Thus, LINC01770 may serve as a key molecular bridge facilitating this aggressive phenotype.

A particularly intriguing finding in our study is that, despite the increase in EMT and stemness induced by LINC01770 overexpression, this did not translate into enhanced in vitro chemoresistance. Although the literature regarding the function of LINC01770 remains scarce, it has been reported that LINC01770 overexpression serves as a poor prognostic marker and promotes cellular invasion in nasopharyngeal carcinoma [9]. However, it sensitizes these cells to ferroptotic cell death by stabilizing *TEAD1* via sponging miR-615-5p and miR-1293, which subsequently favors the expression of Acyl-CoA synthetase long-chain family member 4 (*ACSL4*) and Transferrin Receptor 1 (*TFRC*) [9]. This molecular behavior could either render these cells more susceptible to specific chemotherapeutic agents or fail to trigger downstream survival pathways. Interestingly, the YAP/TAZ/TEAD1 axis has been documented to operate both cooperatively with and independently of the canonical Wnt/β-catenin pathway [24,25]. While the activation of canonical Wnt signaling is widely known to drive multidrug resistance in cancer cells, for example by transcriptionally upregulating ATP-binding cassette (ABC) transporters [26], it could be possible that the activation of the YAP/TAZ/TEAD1 pathway occurs independently of the canonical Wnt/β-catenin pathway in this context. This molecular uncoupling could explain why LINC01770 overexpression selectively drives aggressive traits without altering the resistance to 5-fluorouracil and cisplatin. Tumorigenic capacity is another feature of malignant transformation and tumor aggressiveness [27]. Our results showed that LINC01770 overexpression resulted in a significant increase in colony size without affecting colony number (Figure 4). This observation suggests that LINC01770 may preferentially enhance proliferative capacity under anchorage-independent conditions rather than the initial clonogenic potential. Interestingly, despite promoting stem-like features and aggressive behavior, LINC01770 overexpression did not confer increased resistance to chemotherapeutics such as 5-FU and cisplatin (Figure 6). This finding underscores the functional heterogeneity of cancer stem-like traits and indicates that LINC01770-driven stemness may be uncoupled from classical drug resistance mechanisms. These results suggest that LINC01770 may regulate specific subsets of stemness-related pathways that primarily influence metastasis and tumorigenesis processes rather than therapy response.

Our bioinformatics analysis identified that hsa-miR-335-5p is a target of LINC01770, a finding that was functionally validated by the subsequent upregulation of OCT4 upon LINC01770 overexpression (Figure 7). Consistent with the established literature confirming that miR-335-5p directly targets and represses OCT4 [28,29,30], we propose a model wherein LINC01770 functions as a competing endogenous RNA. By sponging miR-335-5p, LINC01770 effectively relieves OCT4 from post-transcriptional suppression. This regulatory circuit is particularly significant given that miR-335-5p has been previously characterized as a potent tumor suppressor in various malignancies, often lost during the transition to a metastatic phenotype [13,31]. Furthermore, it is critical to note that the proposed interaction between LINC01770 and miR-335-5p relies on computational prediction algorithms. While the downstream upregulation of OCT4 mRNA upon LINC01770 overexpression aligns with the competitive endogenous RNA hypothesis, direct functional validation such as luciferase reporter assays was not conducted. Consequently, this specific regulatory axis must be interpreted as a putative mechanism. Further biochemical characterization will be essential in future studies to formally validate the direct binding and sponging kinetics of LINC01770 in BC cellular models. In addition, it should be noted that our characterization of stemness-associated markers was conducted strictly at the mRNA level. While these transcriptomic profiles and their associated functional phenotypes are robust, the lack of direct validation at the protein level represents a limitation when interpreting the underlying molecular signaling.

Finally, by sequestering miR-335-5p, LINC01770 increased the expression of OCT4, a core transcription factor essential for maintaining the cancer stem cell pool [32,33]. Consequently, the expansion of CD133^+^/CD44^+^ populations and the induction of EMT markers provide a mechanistic explanation for the heightened aggressiveness observed in our models.

## 4. Materials and Methods

### 4.1. Cell Line Culture

The BC cell lines used in this study were obtained from the institutional cell bank of the Cell Communication and Cancer Program at Universidad del Desarrollo, Santiago, Chile. These cell lines were originally sourced from the American Type Culture Collection (ATCC, Manassas, VA, USA). MCF-7, MDA-MB-231, ZR-75-1, and T-47D cells were grown in DMEM high-glucose medium (Cat. No. 10-013-CM, Corning Inc., Corning, NY, USA) supplemented with 10% fetal bovine serum (FBS) (Cat. No. 12133C, Merck, St. Louis, MO, USA) and 1% penicillin/streptomycin (Thermo Fisher Scientific, Waltham, MA, USA). All cell lines were maintained at 37 °C with 5% CO_2_.

### 4.2. Lentiviral Transduction

Lentiviral transduction was conducted as per Perez-Moreno et al. [34]. Briefly, the full-length LINC01770 cDNA sequence was cloned into the multicloning site (MCS) of pLVX-PURO plasmid (Clontech, Mountain View, CA, USA). Lentiviral particles were produced by transfecting Lenti-X 293 T cells (Clontech, Mountain View, CA, USA) with Lenti-X packaging single shots (Clontech, Mountain View, CA, USA) alongside 7 µg of either the LINC01770-expressing lentiviral plasmid or the empty vector control. At 48 h post-transfection, the lentiviral supernatants were harvested and cleared through a cellulose acetate filter with a pore size of 0.45 μm (Corning Inc., Corning, NY, USA). Successful viral production was confirmed using Lenti-X GoStix Plus (Clontech, Mountain View, CA, USA). For the cell transduction, MCF-7 and MDA-MB-231 cells were seeded at a density of 6 × 10^6^ cells/well in a 100 mm culture dish and transduced with the recombinant lentiviruses at a multiplicity of infection (MOI) of 5 under standard growth conditions. At 24 h post-transduction, the stable cells were selected with 7.5 µg/mL puromycin (Invivogen, San Diego, CA, USA) for 72 h. Cells were examined with a Nikon H600L Microscope (Nikon, Melville, NY, USA).

### 4.3. RT-qPCR

Total RNA was extracted using the EZNA Total RNA Kit I (Omega Bio-tek, Norcross, GA, USA) and treated with DNase I (Omega Bio-tek, Norcross, GA, USA). Reverse transcription was performed using the AffinityScript QPCR cDNA Synthesis Kit (Agilent Technologies, Santa Clara, CA, USA) according to the manufacturer’s directions. Quantitative real-time PCR was conducted on an AriaMX Real-time PCR system (Agilent Technologies, Santa Clara, CA, USA) using Brilliant SYBR Green PCR Master Mix (Agilent Technologies, Santa Clara, CA, USA). All reactions were performed in triplicate and the relative expression was calculated using the 2^−ΔΔCt^ method with GAPDH as the normalizing gene. The primers used were chosen using the online software Primer3 version 4.1.0 [35], and their sequences are listed in Table 1.

### 4.4. CD133^+^/44^+^ Populations by Flow Cytometry

For CD133^+^/CD44^+^ population analysis, 2 × 10^5^ cells were incubated with 5 μL (0.25 μg) 7-aminoactinomycin D (7-AAD) (BioLegend, San Diego, CA, USA) as a viability marker followed by staining with anti-CD133/APC and anti-CD44/BV-421 (BioLegend, San Diego, CA, USA) antibodies at a ratio of 1 μL/1 × 10^5^ cells. Antibody dilutions were prepared in 200 μL of phosphate-buffered saline (PBS) containing 2% FBS and incubations were performed for 30 min. Unlabeled cells and APC mouse IgG1ƙ and BV-421 mouse IgG1ƙ isotypes (BioLegend, San Diego, CA, USA) were used as controls. Samples were analyzed on a CytoFlex LX Flow Cytometer (Beckman Coulter, Brea, CA, USA) using CytExpert software 2.6 (Beckman Coulter, Brea, CA, USA) at the Universidad del Desarrollo Facility (Instituto de Ciencias e Innovación en Medicina (ICIM), Universidad del Desarrollo, Las Condes, Santiago, Chile).

### 4.5. Soft Agar Assay

For the colony formation assay, 2.5 × 10^3^ cells were suspended in 0.3% Bacto agar (BD Biosciences, Heidelberg, Germany) in DMEM complete medium containing 12.5% FBS. The cell suspension was then poured into 6-well plates containing a bottom layer of 2 mL 0.5% agar. Plates were fed twice a week with 0.2 mL DMEM supplemented with 10% FBS. After 21 days, colonies were stained using 0.005% crystal violet dissolved in 20% methanol for 30 min at room temperature and photographed under a Nikon H600L Inverted Microscope. For colony size analysis, measurable colonies with clearly defined borders were included, whereas staining artifacts, overlapping colonies, and non-specific debris were excluded. Finally, colonies per well were counted and measured using ImageJ 1.54f software (NIH, Bethesda, MD, USA). The relative volume of the colonies was calculated using the formula π/6 × L × W^2^ [36], where L represents the length and W represents the width of the colony. Quantifications were performed from at least three independent experiments, and the same measurement criteria were applied uniformly across all groups.

### 4.6. Cell Migration

Cells (5 × 10^4^) were seeded in Boyden chambers (Corning Inc., Corning, NY, USA) and placed in 24-well plates containing 600 µL DMEM supplemented with 10% FBS. MCF-7 and MDA-MB-231 cells were then incubated for 16 h and 2 h, respectively, at 37 °C and 5% CO_2_. After incubation, cells on the Boyden chambers were fixed and stained with 0.05% crystal violet (Sigma-Aldrich, St. Louis, MO, USA) in 20% methanol (Sigma-Aldrich, St. Louis, MO, USA) for 30 min. Migrated cells were quantified by counting 10 random fields under a Nikon H600L microscope (Nikon).

### 4.7. Cell Invasion

Cells (5 × 10^4^) were seeded into Boyden chambers (Corning Inc., Corning, NY, USA) pre-coated with 40 µg of Geltrex (Gibco, Thermo fisher Scientific, Grand Island, NY, USA) and placed in 24-well plates with 600 µL DMEM supplemented with 10% FBS. MCF-7 and MDA-MB-231 cells were incubated at 37 °C and 5% CO_2_ for 20 h and 6 h, respectively. After incubation, the chambers were fixed and stained with 0.05% crystal violet in 20% methanol for 30 min. Invasive cells were counted under a Nikon H600L microscope (Nikon). Invasive cells were quantified by counting 10 random fields under a Nikon H600L microscope (Nikon).

### 4.8. Viability Assay for Drug Resistance

Cells (1 × 10^4^) were seeded in 96-well plates and cultured in DMEM supplemented with 10% FBS overnight. The medium was then replaced with 100 µL complete medium containing 40 µg/mL of 5-FU for MCF-7 and 200 µg/mL for MDA-MB-231 cells. For cisplatin, 50 µg/mL and 40 µg/mL were used for MCF-7 and MDA-MB-231 cells, respectively. After the drug treatment, the plates were incubated at 37 °C and 5% CO_2_ for 72 h. Then, cells were incubated with 10 µL of MTS reagent (CellTiter 96 AQueous One Solution Cell Proliferation Assay, Promega, Fitchburg, WI, USA) for 3 h at 37 °C. Finally, the absorbance was measured at 490 nm using a Cytation 5 microplate reader (Agilent Technologies, Santa Clara, CA, USA).

### 4.9. In Silico and Bioinformatic Analyses

Bioinformatics analysis of TCGA datasets was performed using the GEPIA2 platform (http://gepia2.cancer-pku.cn/#analysis). Expression data and correlation analyses were retrieved and visualized directly through the web-based interface using default parameters. For mechanistic analyses, computational predictions were performed to evaluate the subcellular localization and molecular interactions of LINC01770. Subcellular distribution was predicted using the iLoc-LncRNA (v2.0) web server (http://lin-group.cn/server/iLoc-LncRNA(2.0)/predictor.php), utilizing its default prediction algorithms. Potential microRNA targets for the core stemness gene OCT4 were screened using the TargetScan database (https://www.targetscan.com) to identify conserved binding sites. Interacting miRNA targets for LINC01770 were identified using the LncBook 2.0 database (https://ngdc.cncb.ac.cn/lncbook/omics/interaction). All bioinformatic database queries and predictive screenings were accessed and performed on 10 March 2026.

### 4.10. Statistical Analysis

Data normality was first evaluated using the Shapiro–Wilk test. For comparison between two groups, the Mann–Whitney U test or *t*-test was used according to the detection in the normality test. For multiple-group comparisons, Kruskal–Wallis followed by Dunn’s post hoc test or ANOVA followed by Tukey’s post hoc test was used according to the detection in the normality test. Data are expressed as the mean ± standard error of the mean (SEM) from at least three independent experiments. Statistical analyses were performed using the GraphPad Prism 5 software (GraphPad, Boston, MA, USA). A *p* value ≤ 0.05 was considered significant.

## 5. Conclusions

Our findings identify LINC01770 as a previously uncharacterized lncRNA that promotes stem-like properties, migratory and invasive behavior, EMT-associated gene expression, and anchorage-independent growth in BC cells. These results position LINC01770 as a novel contributor to tumor aggressiveness and provide a foundation for future studies exploring its potential biological and clinical relevance in BC.

## Figures and Tables

**Table 1:** Primers for RT-qPCR.

Gene	Forward Primer	Reverse Primer
*NANOG*	5′-CATGAGTGTGGATCCAGCTTG	5′-CCTGAATAAGCAGATCCATGG
*OCT-4*	5′-AGGTATTCAGCCAAACGACCA	5′-TCGATACTGGTTCGCTTTCTC
*SOX-2*	5′-AGCTACAGCATGATGCAGGA	5′-GAGTAGGACATGCTGTAGGT
*SLUG*	5′-TCATCTTTGGGGCGATGAG	5′-CAATGCATGGGGGTCTGAA
*SNAI1*	5′-CTTCCAGCAGCCCTACGAC	5′-GACAGATCCCAGATGAGCA
*TWIST*	5′-GCATCACTATGGACTTTCTCTATT	5′-GCCAGTTTGATCCCAGTATT
*GAPDH*	5′-GAGTCAACGGATTTGGTCGT	5′-GACAAGCTTCCCGTTCTCAG
*LINC01770*	5′-GCAAGCTGTACCACTTCGAG	5′-GACCAGCTCACAGAGGAACA

## Data Availability

The original contributions presented in the study are included in the article/Supplementary Materials; further inquiries can be directed to the corresponding author.

## Supplementary Materials

The following supporting information can be downloaded at https://www.mdpi.com/article/10.3390/ph19071039/s1, Figure S1: Proliferation rates in MCF-7 and MDA-MB-231 cell lines; Figure S2: Concentration curves of 5-FU and cisplatin for MDA-MB-231 and MCF-7 breast cancer cells.
