# Supplementary material for: LINC01770 Is Associated with Stem-like Features and Aggressive Traits in Breast Cancer Cells Through a Putative miR-335-5p/OCT4 Axis
Source: Pharmaceuticals (Basel). 2026 Jul 3;19(7):1039. doi: 10.3390/ph19071039 (PMC13416054; doi:10.3390/ph19071039)
Supplement: Supplementary file 1 [file pharmaceuticals-19-01039-s001.zip › pharmaceuticals-4319967-supplementary.pdf]

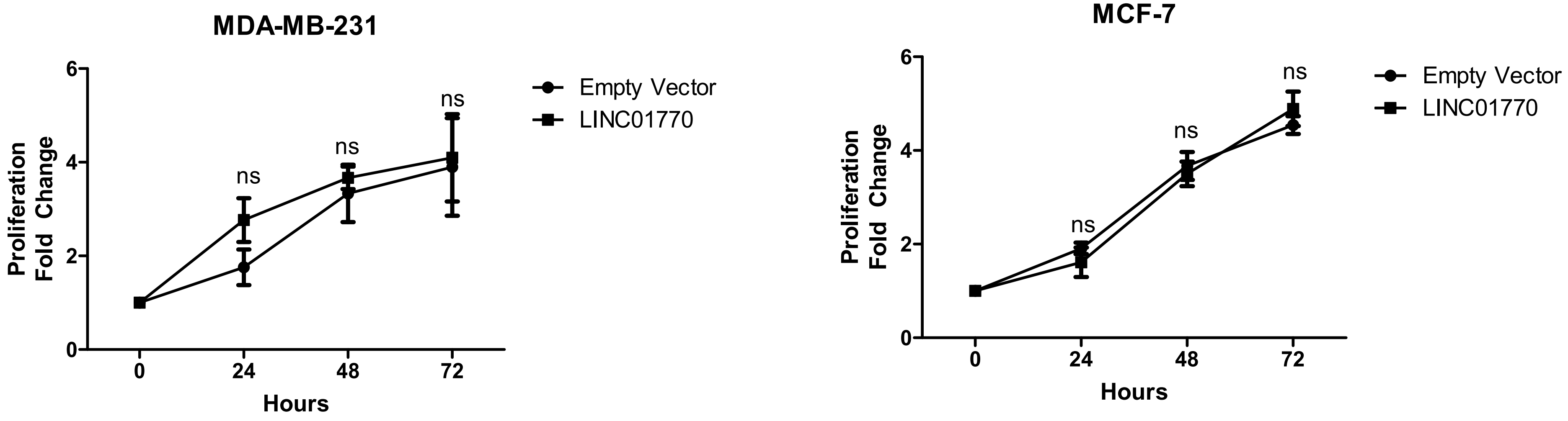

Figure S1. Proliferation rates in MCF-7 and MDA-MB-231 cell lines. Cell proliferation was monitored until 72 h under standard culture conditions to assess the growth kinetics of both breast cancer models by trypan blue assay. Data represents the mean of three independent experiments. ANOVA and the Tukey post-test were used. ns: not significant.

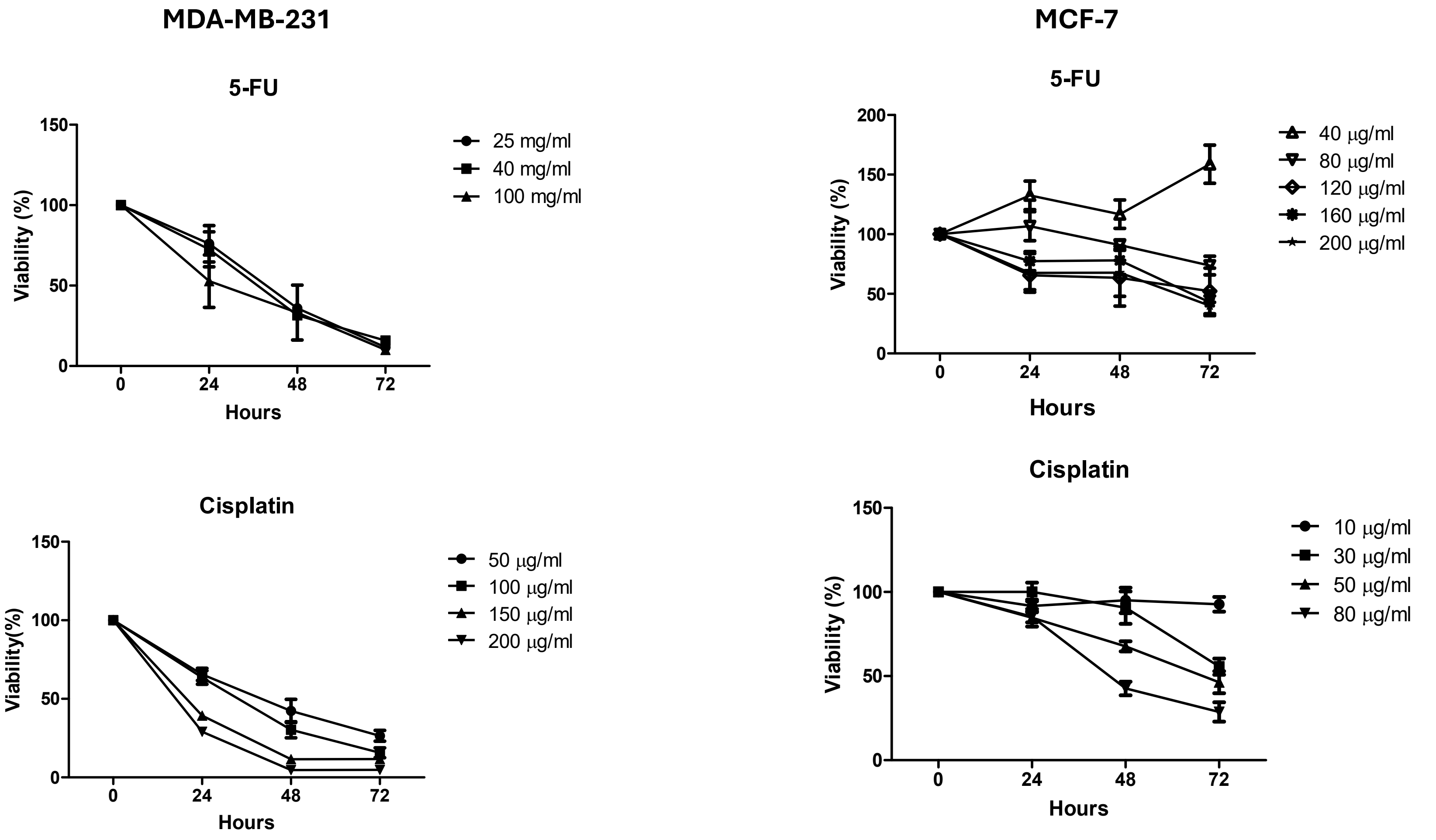

Figure S2. Concentration curves of 5-FU and cisplatin for MDA-MB-231 and MCF-7 breast cancer cells. Cell viability was evaluated by MTS assay in different times (0, 24, 48 and 72 hours) with an exposure to a gradient of 5-fluorouracil (5-FU) and cisplatin. Data represents the mean of three independent experiments.
